# Non-fatal strangulation and COVID-19 common symptoms and signs: considerations for medical and forensic assessment

**DOI:** 10.1007/s12024-022-00460-x

**Published:** 2022-02-09

**Authors:** Lyndal Bugeja, Janine Rowse, Nicola Cunningham, Jo Ann Parkin

**Affiliations:** 1 Department of Forensic Medicine, Monash University, 65 Kavanagh Street, Southbank, Melbourne, VIC Australia; 2 Department of Clinical Forensic Medicine, Victorian Institute of Forensic Medicine, 65 Kavanagh Street, Southbank, Melbourne, VIC Australia

**Keywords:** COVID-19, Non-fatal strangulation, Sexual assault, Physical assault, Interpersonal violence, Injuries, Forensic medicine

## Abstract

Emerging evidence suggests that an onset or escalation of interpersonal violence has been occurring during the COVID-19 pandemic, particularly among persons in intimate or familial relationships. Strangulation (or neck compression) is a common form of interpersonal violence and can result in serious adverse health outcomes, including death. The identification and attribution of injuries from non-fatal strangulation are complex, as there may be an absence of external signs of injury and their appearance may be delayed by many days. There is a heavy reliance on clinician identification of ‘red flag’ symptoms and signs, the presence of which necessitates urgent further assessment. Additional challenges arise when acute non-fatal strangulation symptoms and signs are shared with other clinical conditions. In such cases, differentiating between the conditions based on the symptoms and signs alone is problematic. We present the diagnostic challenges faced when conducting forensic assessments of COVID-19-positive and suspected COVID-19 (S/COVID) patients following allegations of non-fatal strangulation in the setting of physical and sexual assaults. The implications of shared symptoms and signs, for forensic clinicians, primary healthcare, and emergency practitioners, as well as other frontline service providers, are discussed.

## Introduction

Coronavirus disease-2019 (COVID-19) is a contagious illness caused by severe acute respiratory syndrome-coronavirus-2 (SARS-CoV-2) [1]. In addition to the dramatic loss of human life that has occurred across the globe, the COVID-19 pandemic has also resulted in significant economic and social disruption. An issue of particular concern is an increased incidence of interpersonal violence, specifically domestic and family violence, gender-based violence, and child abuse and neglect [2]. Factors identified that contribute to the onset or escalation of interpersonal violence during the COVID-19 pandemic include as follows: increased pandemic-related stress (for example, financial or employment insecurity); harmful alcohol use; forced cohabitation with an abuser during lockdown periods; no or limited avenues of opportunity to seek help; and diversion of police resources to manage public order [3].

An online survey of 15,000 Australian women reported that the initial phase of the COVID-19 pandemic coincided with the onset of domestic violence for many women [4]. One-third of women that experienced physical or sexual violence during the period February 2020 to May 2020 reported this being the first time their partner had ever been violent towards them [4]. Among women who reported having experienced previous physical or sexual violence from a cohabiting partner prior to February 2020, more than half (53.1%) reported an increase in frequency or severity since the onset of the pandemic [4]. These findings are supported by an Italian study that measured the number of women who accessed anti-violence services during a two-month (2 March to 5 April, and 6 April to 3 May 2020) national lockdown [5]. Compared to the monthly average in 2018, the number of women that accessed anti-violence services increased by 87.7% during the 2 March to 5 April 2020 period, and 82.1% during the 6 April and 3 May 2020 period [5]. A review article that examined 42 publications on the relationship between violence against women and the COVID-19 pandemic reported that ‘stay at home’ policies have increased the problem of violence against women [6]. The study also reported a substantial reduction in intimate partner violence (IPV) victims’ requests for assistance [6].

In many jurisdictions around the world, victims of interpersonal violence are referred by police to forensic clinicians for a forensic medical examination to document evidence in a potential criminal investigation. A key consideration for the forensic clinician is whether a sign or symptom reported by, or observed on, a patient is the result of injury or illness. Establishing the type, mechanism, cause, and manner of any sign or symptom among patients alleging interpersonal violence is complex and generally requires careful history taking, face-to-face physical examination, photography, and specimen collection.

The COVID-19 pandemic led to changes in how healthcare services were delivered, including the provision of clinical forensic medicine services [7, 8]. Following the World Health Organization’s (WHO) declaration of COVID-19 as a global pandemic in March 2020, a number of jurisdictions [9, 10] incorporated new protocols into their service delivery models and began performing some aspects of their clinical forensic medicine services remotely through Telehealth and other online mediums, including at the Victorian Institute of Forensic Medicine (VIFM) in Australia.

### Examination of COVID-19-positive and S/COVID-19 patients as victims of non-fatal strangulation

This report discusses the shared acute symptoms and signs a of non-fatal strangulation in COVID-19-positive and S/COVID-19 patients referred to the Clinical Forensic Medicine (CFM) service at the VIFM following an allegation of physical or sexual assault. The CFM service at the VIFM is located in Victoria, Australia, and provides a range of expert forensic medical services to Victoria’s population of 6.7 million people. CFM services include assessment of victims and perpetrators of family violence; physical and/or sexual assault examinations and expert opinions; fitness for interview assessments of police detainees; traffic medicine; and advice on clinical aspects of reviewable death investigations for the coroner.

In metropolitan Melbourne, a small team of forensic clinicians from the CFM service at the VIFM conduct sexual assault examinations at five geographically dispersed hospital-based crisis care units and at a multi-disciplinary centre that houses police and counsellors. Forensic medical examinations are conducted in association with Centre Against Sexual Assault (CASA) advocates, who provide patient support, counselling, and aftercare. In contrast, physical assault examinations in Melbourne may be conducted in any of the eight large metropolitan hospitals or their smaller affiliate hospitals, and also within the CFM clinic at the VIFM.

From July 2020, S/COVID and COVID-19-positive patients who were alleged victims of physical assault, sexual assault, and non-fatal strangulation were examined as per the CFM COVID-19 protocols [8]. These protocols have sought to balance DNA contamination minimisation principles with the prevention of viral contamination of forensic samples and packaging, while maintaining staff and patient safety during the pandemic. S/COVID and COVID-19-positive victims of assault were examined in negative pressure rooms where possible and/or dedicated COVID-19 hot zones.

In one such deidentified case, an otherwise healthy female is presented to a tertiary hospital emergency department in Melbourne for medical assessment following an allegation of assault and non-fatal strangulation. She reported a sore throat, experienced pain on swallowing, had marked dysphonia (hoarse voice) and microphonia (abnormally weak voice). As per the COVID-19 testing criteria for symptomatic individuals at the time, she was swabbed for COVID-19. She was then confirmed to be COVID-19-positive and admitted to a specialty COVID-19 ward.

Given the clinical presentation and allegation of assault, the question therefore arose: were her symptoms and signs related to the COVID-19 infection, non-fatal strangulation, or a combination of both? Medical imaging did not identify complications of strangulation, and flexible laryngoscopy was not able to be conducted in this case due to the heightened risks of the procedure in the setting of COVID-19 [11, 12]. Cases such as this raise important issues for the clinical assessment and medico-legal investigation of non-fatal strangulation among S/COVID and COVID-19-positive patients.

## Discussion

The WHO published COVID-19 clinical criteria for a suspected case of the illness: acute onset of fever and cough; or acute onset of three or more of fever; cough; general weakness/fatigue; headache; myalgia; sore throat; coryza; dyspnoea; anorexia/nausea/vomiting; diarrhoea; and altered mental state [13]. In the absence of any other identified cause, the recent onset of anosmia (loss of smell) and/or ageusia (loss of taste) is included in the clinical criteria of a probable COVID-19 case. In addition to the WHO criteria, findings from medical and empirical research have identified other symptoms and signs of COVID-19 from observational studies, and self-reported studies of patients with mild, moderate, and severe forms of the illness. These include the following: chest pain or pressure; dysphonia; malaise; nasal congestion; and rhinorrhoea [14–17].

Research has also been crucial in identifying the symptoms and signs of non-fatal strangulation, which is defined as external compression of a person’s neck and/or upper torso in a manner that inhibits that person’s airway or the flow of blood into or out of the head [18]*.* This research has informed the development of non-fatal strangulation toolkits and reference guides for forensic clinicians, investigators, and prosecutors on the ‘red-flag’ symptoms and signs of non-fatal strangulation [19, 20].

**Fig. 1:**
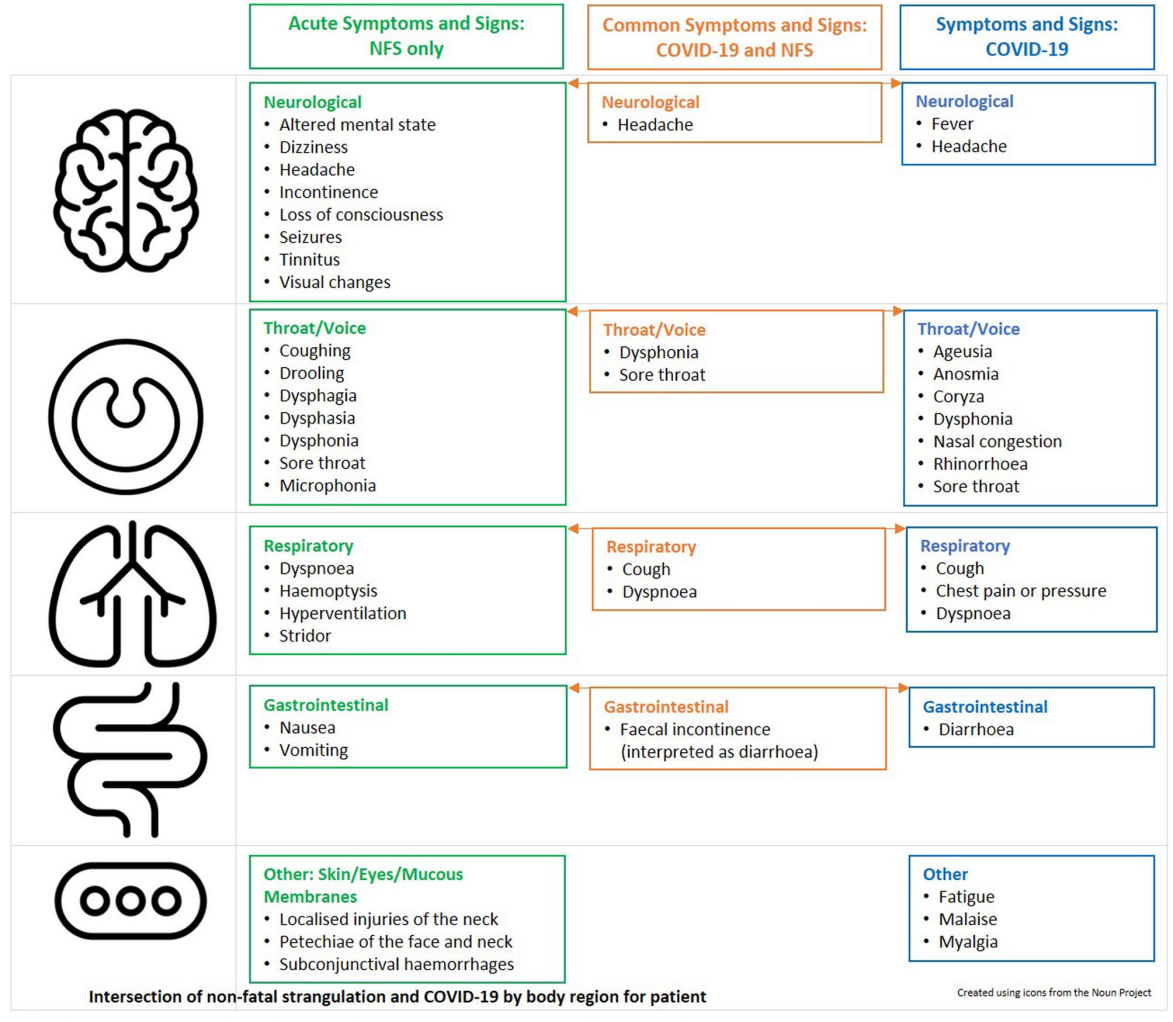
Intersection of non-fatal strangulation and COVID-19 by body region for patient

When the acute symptoms and signs of non-fatal strangulation and COVID-19 are considered together, there is some commonality in the neurological, otolaryngological (mouth/nose/throat/voice), respiratory, and gastrointestinal findings. In the patient described above, there were shared otolaryngological (throat/voice), respiratory symptoms, and signs of non-fatal strangulation and COVID-19: dysphonia and sore throat (Fig. 1).

Supporting an allegation of non-fatal strangulation with forensic evidence is challenging. Identifying the acute symptoms and signs of non-fatal strangulation has implications for the medico-legal findings and the utility of these findings for a criminal investigation. The difficulty often arises in the prosecution of non-fatal strangulation cases as the victims frequently present with an absence of visible signs of injury.

During the COVID-19 pandemic, additional challenges have arisen for the assessment of victims who present with a history of attempted strangulation; an absence of visible external signs of injury to the neck (e.g., bruising, abrasions); the presence of symptoms and signs that are shared with both non-fatal strangulation and COVID-19; and who test positive for COVID-19. The COVID-19 diagnosis can become the overwhelming and distracting finding at the expense of identification of potential non-visible injuries of non-fatal strangulation. It is of crucial importance that frontline healthcare providers and forensic clinicians are aware of the shared acute symptoms and signs between non-fatal strangulation and COVID-19, as failure to consider either diagnosis may result in catastrophic outcomes. The recognition of a COVID-19 diagnosis has significant implications for the mode of delivery of healthcare services (i.e., in COVID-19 designated wards), and subsequent forensic assessment, using modified COVID-19 protocols [8]. Similarly, failure to recognise red flags in a symptomatic COVID-19-positive patient alleging an assault with attempted strangulation risks missing the development of life-threatening complications of neck compression.

The absence of symptoms and signs following neck compression is well recognised. A retrospective review of 300 strangulation cases found that 67% reported no symptoms to police and 50% had no visible signs [21]. A study by Zilkens et al. of sexual assault victims found that almost a quarter of women alleging non-fatal strangulation had neither signs nor symptoms [22]. In cases where symptoms do occur the time of onset, and delays in presentations to healthcare services or police, is a further complicating factor. The development of non-fatal strangulation symptoms and signs may be subtle or delayed by many days [18], making it difficult for the healthcare provider or forensic clinician to identify an injury, particularly, if the patient was assessed immediately following the incident. In cases where the patients themselves do not immediately present to a healthcare service or to police, any symptoms and signs may have resolved before a forensic medical assessment is performed.

A recent European study examined otolaryngological symptoms of 702 patients with mild-to-moderate COVID-19, excluding patients with a potentially confounding history of head and neck trauma. Over a quarter of patients (26.8%) are self-reported experiencing dysphonia, and this was more significant in females [15]. Patients who self-reported dysphonia also reported a higher proportion of systemic symptoms (including cough, chest pain, and headache) compared to non-dysphonic patients. Otolaryngological symptoms and signs are a feature not only of COVID-19 and non-fatal strangulation but also other viral infections. Interestingly, dysphonia is reportedly encountered in less than 20% of common viral infections and may therefore be over-represented in COVID-19 patients [15], which potentially causes a greater frequency in the setting of COVID-19 than with other otolaryngological presentations.

## Conclusion

The COVID-19 pandemic and the associated mechanisms that are being used to reduce community transmission such as lockdown conditions have resulted in growing apprehension of a ‘shadow pandemic’ of domestic violence. Non-fatal strangulation is a serious and frequent feature of intimate partner violence. This case report highlights the implications of assessing victims of non-fatal strangulation during the COVID-19 pandemic where there are shared acute symptoms and signs with COVID-19 infection such as dysphonia and sore throat. These red-flag findings have traditionally been interpreted by forensic clinicians as potential evidence of non-fatal strangulation, however, in the context of COVID-19; the presence of a concurrent or alternative diagnosis of COVID-19 infection must be carefully considered.

Given the global experience that the incidence of interpersonal violence, particularly intimate partner violence, has increased during the COVID-19 pandemic, those working in forensic medical and frontline services may encounter a subsequent increase in allegations of non-fatal strangulation. It is imperative that forensic clinicians, primary healthcare, and emergency practitioners, as well as all other frontline service providers, recognise the existence of shared symptoms and signs between non-fatal strangulation and COVID-19 infections, which has important implications for the assessment, medical management, and forensic interpretation of any findings.

## Key points

1. Persons in intimate or familial relationships have experienced an onset or escalation of violence during the COVID-19 pandemic.
2. There are diagnostic challenges when assessing patients with suspected and confirmed COVID-19 alleging non-fatal strangulation as there are shared acute symptoms and signs.
3. We present shared acute symptoms and signs using a case example of a patient assessed by forensic medicine physicians from the Victorian Institute of Forensic Medicine in Australia.

## Data Availability

Not applicable.
